# Active surveillance for the safety and effectiveness of health products for COVID-19: a scoping review

**DOI:** 10.3389/fphar.2026.1752571

**Published:** 2026-04-02

**Authors:** Said Yousef, Zemin Bai, Shannon E. Kelly, Becky Skidmore, George Wells

**Affiliations:** 1 Cardiovascular Research Methods Centre, University of Ottawa Heart Institute, Ottawa, ON, Canada; 2 School of Epidemiology and Public Health, University of Ottawa, Ottawa, ON, Canada; 3 Independent Information Specialist, Ottawa, ON, Canada

**Keywords:** active surveillance, effectiveness, safety, SARS-CoV-2, treatment

## Abstract

Urgent response to the coronavirus disease 2019 (COVID-19) pandemic necessitated rapid implementation of experimental, re-purposed, or off-label treatment strategies, which can be monitored *via* active surveillance (AS). Using a scoping review methodology, we summarized AS systems used to assess the safety and effectiveness of health products for COVID-19 treatment. We searched electronic databases (MEDLINE, Embase, Web of Science, and Cochrane Library), global regulatory agency websites, and registries, and implemented alerts until August 2022. The records with data source, active data access, and timely reporting that were applied in COVID-19 treatment were considered eligible. Fifteen publications to describe 13 AS systems were identified from a total of 9,183 literature records. Six systems were designed for safety, one for effectiveness, three for both, and three provided descriptive treatment data. Eleven systems were repurposed, and two were created during the pandemic. Twelve were initiated for COVID-19 in 2020, and one existing system was applied for a safety study in 2022. Various data sources, technical tools, procedures, and study designs were applied to provide active data access and timely analyses of safety and effectiveness of antivirals, antibiotics, hydroxychloroquine, corticosteroid, and others used for COVID-19. The COVID-19 pandemic accelerated the development and repurposing of AS to address urgent challenges in evaluating the safety and effectiveness of therapeutic interventions. In this scoping review, we highlight the essential role of AS in generating timely real-world evidence, supporting clinical and regulatory decision-making, and contributing to healthcare system resilience. The findings also reveal gaps in transparency and standardization, highlighting the need for integrated, ethically governed AS infrastructures that support data privacy, public trust, and timely evidence generation for future public health emergencies.

## 1 Introduction

Coronavirus disease 2019 (COVID-19), caused by severe acute respiratory syndrome coronavirus 2 (SARS-CoV-2), has become a global health crisis, with more than 776 million confirmed cases and approximately 7 million deaths reported worldwide until now (Cascella et al., 2022; World Health Organization, 2024a; World Health Organization, 2024b). Although most individuals experience mild to moderate respiratory symptoms, older adults and those with underlying health conditions face a higher risk of severe illness and mortality (World Health Organization, 2024a). The initial phase of the pandemic posed an unprecedented challenge as no established pharmaceutical products were proven safe and effective for treating this novel disease (World Health Organization, 2020; Rodriguez-Guerra et al., 2021).

In the absence of specific treatments, the medical community turned to off-label use of existing medications, experimental therapies, and drug repurposing under emergency use conditions with close monitoring (Cascella et al., 2022; World Health Organization, 2020). Notable examples include antiviral drugs such as Paxlovid, anti-SARS-CoV-2-neutralizing antibodies such as convalescent plasma and bamlanivimab/etesevimab, anti-inflammatory drugs such as dexamethasone, and immunomodulator agents such as tocilizumab (Cascella et al., 2022). However, uncertainties persist regarding the efficacy of these treatments against emerging SARS-CoV-2 variants and subvariants (World Health Organization, 2022). Amid the evolving landscape of COVID-19 therapeutics, there is a potential growing role of vitamins C and D due to their antioxidant properties and immunomodulatory effects. Despite this interest, more evidence is needed to support their effectiveness in managing COVID-19 (Rodriguez-Guerra et al., 2021).

The urgent response to the pandemic necessitates the continuous development of new therapeutics, ongoing monitoring, and sequencing of the SARS-CoV-2 virus over time, which is crucial for overcoming the uncertainties and ending the pandemic (Forchette et al., 2021). In this context, pharmacovigilance, particularly active surveillance (AS) methods, emerges as a crucial tool for assessing the safety and effectiveness of health products used to manage COVID-19 (Aronson et al., 2012). Unlike passive surveillance relying on spontaneous reporting, AS involves a systematic and continuous pre-organized process for collecting event reports (ICH Steering Committee, 2011). This method enhances reporting accuracy and the quality of outcome reports, as demonstrated by an observational study and a scoping review (Al-Tajir and Kelly, 2005; Khalili et al., 2020). Although they are traditionally used for adverse event data collection, the application of AS for effective data collection has yet to be explored. Furthermore, real-world data sources are used in larger-scale post-market studies for more timely and robust assessment of potential drug safety signals (Dhodapkar et al., 2022).

As the urgency of the pandemic prompted the rapid implementation of various treatment strategies, in the current scoping review, we aim to identify and assess the AS tools, methods, and approaches implemented for monitoring the safety and effectiveness of health products used to treat COVID-19, with particular attention to special populations such as pregnant women and children.

## 2 Methods

We used a scoping review methodology to identify evidence sources detailing tools, methods, or approaches for actively surveilling health products in COVID-19 treatment. The specific goals were to pinpoint AS tools used in the early pandemic (within 2020) and observe changes in later phases (after 2020). Moreover, we aimed to examine COVID-19 therapeutics-related scientific hypotheses and assess reported data’s reliability and validity. In this review, we followed the Arksey and O'Malley methodological framework (Arksey and O’Malley, 2005), the methodology manual published by the Joanna Briggs Institute (The Joanna Briggs Institute, 2015), and the PRISMA-ScR checklist (Tricco et al., 2018).

In consultation with content experts and knowledge users, in this review, we followed a predetermined study protocol (Bai et al., 2023).

### 2.1 Information sources and search strategy

With input from the review team and content experts, an information specialist developed a comprehensive literature search strategy, peer-reviewed by a second independent research librarian using the Peer Review of Electronic Search Strategies (PRESS) checklist (McGowan et al., 2016). The strategy covered the databases of Ovid MEDLINE, Embase, Cochrane Central Register of Controlled Trials, Cochrane Database of Systematic Reviews, and Web of Science. A combination of controlled vocabulary and free-text terms related to COVID-19, pharmaceutical preparations, and post-marketing product surveillance was implemented. The search, executed on May 13, 2021, and the search alerts continued until August 20, 2022. Pre-print servers (medRxiv and Research Square), COVID-END inventory, and gray literature searches of regulatory agencies and clinical trial databases were included. The World Health Organization (WHO), Agency for Healthcare Research and Quality, and the National Institutes of Health were also searched. The search results were downloaded and deduplicated in Endnote version 9.3.3 (Clarivate) in preparation for screening. The final search strategy is available along with the protocol registration.

### 2.2 Eligibility

Eligibility criteria were established for the population, intervention, comparator, outcome, and study design (PICOs), as follows:

#### 2.2.1 Population

Individuals with suspected or confirmed acute COVID-19 infection, including “long-haulers” of any age or sex who received health products as a therapeutic intervention, including pharmaceuticals, biologics, and natural health products, regardless of their regulatory approval status, were eligible.

#### 2.2.2 Intervention

All AS methods, tools, or approaches with a population-level focus informing safety or effectiveness assessments of health products were eligible.

We applied the broad definition of Aronson et al. (2012), “a form of non-interventional public health research, consisting of a set of processes for the continued systematic collection, compilation, interrogation, analysis, and interpretation of benefits and harms.” The method includes the proposed or established procedure or approach for AS that could be followed, repeated, or reproduced, especially a systematic or established procedure. It could also include techniques, systems, practices, routines, methods of working, formulas or algorithms, processes, or mechanisms. The tool that enabled or facilitated AS observation, measurements, or data collection were defined as a device, product, or instrument (e.g., product or patient registries, electronic medical records, and administrative databases). Each intervention collected was adjudicated by examining the data source, descriptions of how and who to access data, and timeliness of the data or reports. Only interventions with active data access and timely reports were considered eligible.

#### 2.2.3 Comparators

Any comparator or no comparator was eligible.

#### 2.2.4 Outcomes

The methodological outcomes of interest were broadly defined as the characteristics and capabilities of AS methods or tools and the validity and reliability of the data captured by these methods or tools.

#### 2.2.5 Study designs

All study designs were considered eligible if they reported AS methods and provided population-level active data access and reporting.

Any records not reporting relevant AS methods; reports of passive surveillance, public health surveillance (e.g., outbreak investigation), or individual therapeutic response; studies on social media and crowd-sourced interventions; and non-human or *in vitro* studies were excluded. Vaccines, medical devices, and digital health products were beyond the scope of this review.

### 2.3 Study selection

Three pilot screening exercises were sequentially conducted by two reviewers to accommodate the broad scope of study designs and the limited information on AS methods in abstracts.

Two reviewers independently screened all retrieved titles and abstracts. Given a high inclusion rate of 25%, the full-text screening approach was modified as one reviewer initiated the screening and another reviewer verified.

Website checks were conducted for additional details on AS approaches when primary publications lacked information. Discrepancies at both selection levels were discussed to reach a consensus between the two reviewers. Otherwise, a third independent reviewer was involved. All selection phases were conducted using Covidence web-based data management software for systematic review management (Covidence, 2025).

### 2.4 Information items and information abstraction

One reviewer extracted information on AS system components, with a second reviewer independently verifying collected data. When studies lacked information, reviewers referenced related publications or websites. Summarized details were presented in evidence tables. Extraction focused on attributes including surveillance scope, data collection and processes, and method/tool evaluation (e.g., simplicity, flexibility, and acceptability). Information on surveillance system structure, partnerships, funding, considered data, and patient populations was also obtained. Information on publication/conduct dates was collected for the classification into early or later pandemic phases, along with PICOS elements, settings, and countries. The rapidness of implementation, reported barriers/enablers, and study hypotheses were also recorded.

### 2.5 Critical appraisal

No risk of bias or quality appraisal was conducted in the included studies. Author-reported limitations and strengths were extracted, and additional informal appraisal was provided from a methodological perspective. This approach is consistent with the methods manual published (The Joanna Briggs Institute, 2015) and a health-related scoping review (Tricco et al., 2016).

### 2.6 Synthesis

Result synthesis focused on providing a descriptive summary of all reported AS methods and tools, including evaluations of validity and reliability. Findings from the included studies are presented in detailed tables.

## 3 Results

**FIGURE 1:**
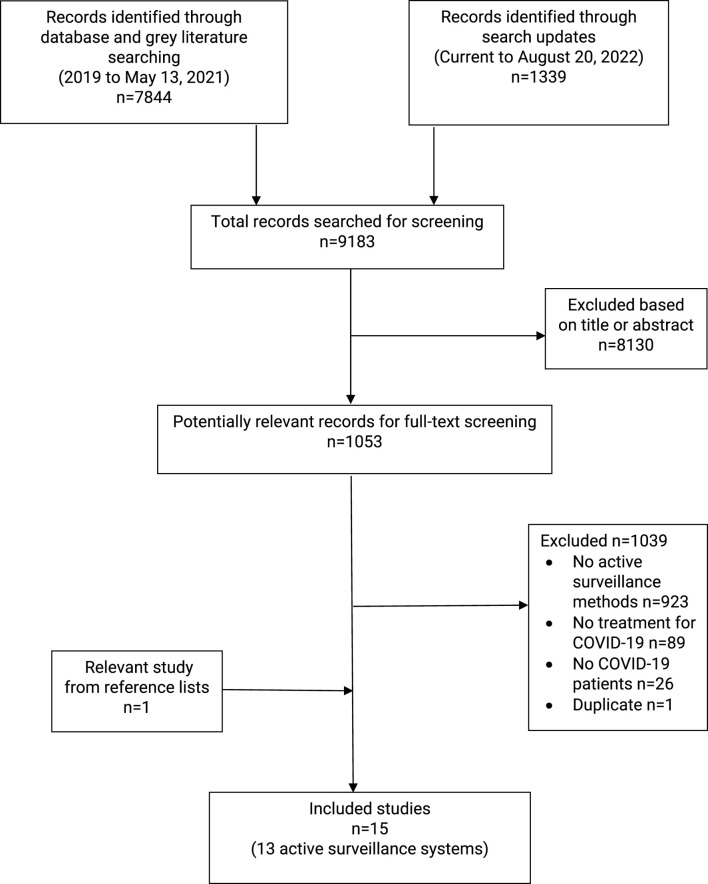
Flowchart of literature screening.

A total of 9,183 records were initially identified through the literature search. After screening titles and abstracts, 1,053 records were screened for full-text screening, including 14 studies (Chouchana et al., 2021; Gerard et al., 2021; Romani et al., 2021; Gerard et al., 2020; Falcao et al., 2021; Darko et al., 2020; Skalafouris et al., 2021; WHO, 2019; Cocoros et al., 2021; Simpson et al., 2020; de Lusignan et al., 2020; Burke et al., 2021; Gale et al., 2021; Mitchell et al., 2021) and an additional study (Knight et al., 2020) identified from a reference list. These 15 studies supported 13 active surveillance-related interventions (Figure 1). They were described as six AS systems (Falcao et al., 2021; Darko et al., 2020; Skalafouris et al., 2021; Cocoros et al., 2021; Gale et al., 2021; Knight et al., 2020), two AS programs (Chouchana et al., 2021; Gerard et al., 2021; Mitchell et al., 2021), one relevant informatics digital hub (de Lusignan et al., 2020), one platform (Burke et al., 2021), one network (Romani et al., 2021; Gerard et al., 2020), one surveillance (Simpson et al., 2020), and one monitoring method (WHO, 2019). Hereafter, the interventions included are referred to as “AS systems.” Furthermore, they were used to evaluate the safety and effectiveness of health products for COVID-19 treatment (Table 1). During the full-text review, 1,039 records were excluded, primarily due to the absence of AS methods or tools (n = 923), no treatment for COVID-19 (n = 89), no COVID-19 participants (n = 26), or duplicate records (n = 1) (Figure 1).

**TABLE 1:** Summary table of the 13 active surveillance (AS) systems.

AS systems /country	Year established	Date applied for COVID-19	Type of data source	Data accessing	Timeliness of reporting	Type of the example study	Patient with COVID-19	Health products for COVID-19	Main outcomes of interest
AS on safety									
WHO–PIDM program (Chouchana et al., 2021; Gerard et al., 2021) International	1968	January of 2020	VigiBase ICSRs	The WHO tools, VigiLyze for signal detection, and VigiAccess for public view	Quarterly reported and continuously updated	Retrospective case–noncase study	Case safety reports in VigiBase	REM	Acute kidney injury (AKI), tubular necrosis, and kidney disorder
French pharmacovigilance network (Romani et al., 2021; Gerard et al., 2020) France	1985	March of 2020	FPVD ADR reports	Platforms for signal detection or signal replication	Early detection of HCQ CADRs	Retrospective comparative observational study	Post-marketing CADRs associated with HCQ	HCQ	Repolarization and ventricular rhythm disorders, sinus bradycardias, and incidence of CADRs
NPS system (Falcao et al., 2021) Portugal	1992	March of 2020	Patients’ chart review ADR reports	Assessment of causality and evaluation of the actions triggered	ADR reports sent to INFARMED as soon as possible	Prospective observational study	Patients treated with either REM or HCQ alone or in combination with other medications	REM HCQ	ADR cumulative incidence, drug discontinuation, and causality assessment
SafetyWatch system (Darko et al., 2020) Ghana	2001	April of 2020	Ghanaian pharmacovigilance database ADR reports	Assessment of causality and expectedness of ADRs	Daily follow-ups and weekly updates	Prospective study of individual case safety reports	Patients treated with any medication	LPV/r Doxy, AZM, and HCQ mPSL	ADRs, serious ADRs, and causality assessment of ADRs
PharmaCheck system (Skalafouris et al., 2021) Switzerland	2020	February of 2020	Hospital’s EMR	Measurement of alerts and triggers and recommendations to prescribers	Twice daily	Prospective observational study	Hospitalized patients with current LPV/r, a history of HCQ, or both	LPV/r HCQ	Alert and triggers, recommendations for therapeutic optimization or additional monitoring
CEM method (WHO, 2019) International	No year reported (existed)	March of 2022	Questionnaire Medical records	Online database for data analysis, informing safety concerns, and sharing study results	Monthly interim analysis, reporting SAEs in real time, and full report after database lock	Observational prospective single-arm cohort study (protocol)	Patients treated by MOV in low- and middle-income countries	MOV	AEs, SAEs, maternal, and perinatal outcomes
AS on safety and effectiveness									
FDA’s Sentinel System (Cocoros et al., 2021) US	2009	April of 2020	US-based EHRs Administrative claims	Code list and analytic programs for public, and EHR data accessible for Sentinel partners	Using near-real-time claims data and updated data every 2 weeks	Surveillance report of COVID-19	Hospitalized patients (including pregnant women and children)	REM DEX LMWH MABs	ICU stay, death, MV, or ECMO
EAVE II surveillance (Simpson et al., 2020) Scotland	2015 (EAVE)	June of 2020 (EAVE II)	Scottish national linked databases	Metadata produced available to Health Data Research United Kingdom Gateway	Undertaking timely analyses	Prospective observational cohort study (protocol)	Residents in Scotland registered with a general practice	Existing or new therapies and ABX	Effectiveness and safety of new or existing therapies, and antimicrobials
ORCHID digital hub (de Lusignan et al., 2020) United Kingdom	1967	2020	Computerized medical records	Hub membership for metadata repositories and online application system for study-ready datasets or custom datasets	Weekly updates on the latest surveillance and research findings, and providing near-real-time data	Protocol to develop the extended COVID-19 surveillance platform	Patients with suspected and confirmed COVID-19	Concurrent medication that may influence COVID-19 outcomes (e.g., ACEI)	Hospital and ICU admission, MV, death, and ABX consumption
AS on effectiveness									
REACT COVID-19 platform (Burke et al., 2021) United Kingdom	2020	March of 2020	Hospital’s EMR Clinical notes	Encrypted web service for researchers to access study data	Data captured in real time and backed up weekly	Prospective observational study (ongoing)	Patients under the care of UHSFT	Antivirals ABX CortACEI/ARB	Death, invasive ventilation COVID-19 complications
AS with descriptive treatment data									
BPSU system (Gale et al., 2021) United Kingdom	1985	April of 2020	E-reporting card Questionnaire	Early signals identified immediately available to the related governments or organizations	Weekly basis to identify early signals from the surveillance to support police and clinical decision-making	National prospective cohort study (ongoing)	Hospitalized babies in the first 28 days of life in the United Kingdom	Antivirals ABX Cort AAD Ig	Length of stay in hospital, transferred to another hospital, and Death
CNISP program (Mitchell et al., 2021) Canada	1994	March of 2020	Standardized case report form Questionnaire	Aggregate reports and national reports to all CNISP hospitals	Real-time hospital-based surveillance data, weekly aggregated report, and monthly descriptive report	Prospective sentinel surveillance (ongoing)	Any patients admitted to a CNISP hospital within 14 days of a positive test	Antivirals ABX HCQ Cort	ICU admission, MV, death, and Discharged
UKOSS system (Knight et al., 2020) United Kingdom	2005	March of 2020	E-reporting card E-data collection form	Data analyzed and reported on newsletters, annual reports, and peer-reviewed publications	Quarterly newsletters, annual reports, and monthly reviewed guidance for pregnant women	Prospective national population-based cohort study (ongoing)	Hospitalized pregnant women in the United Kingdom	Antivirals Cort	CCU admission, maternal death, neonatal death, fetal loss, and infant infection

AAD, anti-arrhythmic drug; ABX, antibiotics; ACEI/ARB, angiotensin-converting enzyme inhibitor/angiotensin receptor blocker; ADR, adverse drug reaction; AE, adverse event; AS, active surveillance; AZM, azithromycin; BPSU, British Paediatric Surveillance Unit; CADRs, cardiac adverse drug reactions; CCU, critical care unit; CEM, cohort event monitoring; CNISP, Canadian Nosocomial Infection Surveillance Program; Cort, corticosteroid; DEX, dexamethasone; Doxy, doxycycline; EAVE, early estimation of anti-viral and vaccine effectiveness; EAVE II, early pandemic evaluation and enhanced surveillance of COVID-19; ECMO, extracorporeal membrane oxygenation; EHR, electronic health record; EMR, electronic medical record; FDA, Food and Drug Administration; FPVD, French PharmacoVigilance Database; HCQ, hydroxychloroquine; HUG, Geneva University Hospital; ICSR, individual case safety report; ICU, intensive care unit; Ig, immunoglobulin; LMWH, low-molecular-weight heparin; INFARMED, Portuguese National Authority for Medicines and Health Products; LPV/r = lopinavir/ritonavir; MABs, monoclonal antibodies; MOV, molnupiravir; mPSL, methylprednisolone; MV, mechanical ventilation; NPS, National Pharmacovigilance System; ORCHID, Oxford Royal College of General Practitioners Clinical Informatics Digital Hub; PIDM, Programme for International Drug Monitoring; REACT, Research Evaluation Alongside Clinical Treatment; REM, remdesivir; SAE = serious adverse event; UHSFT, University Hospital Southampton NHS foundation trust; UK, United Kingdom; UKOSS, United Kingdom Obstetric Surveillance System; US, United States; VigiBase, WHO global database of individual case safety reports; WHO, World Health Organization.

### 3.1 General characteristics of active surveillance systems

Eleven of the identified 13 AS systems were previously established between 1967 and 2015 for other diseases such as influenza (Chouchana et al., 2021; Gerard et al., 2021; Romani et al., 2021; Gerard et al., 2020; Falcao et al., 2021; Darko et al., 2020; WHO, 2019; Cocoros et al., 2021; Simpson et al., 2020; de Lusignan et al., 2020; Gale et al., 2021; Mitchell et al., 2021; Knight et al., 2020). Ten of them (Chouchana et al., 2021; Gerard et al., 2021; Romani et al., 2021; Gerard et al., 2020; Falcao et al., 2021; Darko et al., 2020; Cocoros et al., 2021; Simpson et al., 2020; de Lusignan et al., 2020; Gale et al., 2021; Mitchell et al., 2021; Knight et al., 2020) were repurposed for COVID-19 surveillance in 2020; one existing system cohort event monitoring (CEM) method (WHO, 2019) was applied in a WHO prospective cohort study for molnupiravir in COVID-19 in 2022. Two systems, the Research Evaluation Alongside Clinical Treatment (REACT) COVID-19 platform at a university (Burke et al., 2021) and the Electronic PharmaCheck system at a hospital (Skalafouris et al., 2021), were specifically developed for COVID-19 surveillance in 2020 (Table 1). Obviously, 12 of the 13 AS systems were implemented for COVID-19 treatment in the early stage of the pandemic within 2020, whereas only one existing system was applied in the later stage after 2020. These 13 systems were developed in eight countries (France, Portugal, Ghana, Switzerland, US, Scotland, United Kingdom, and Canada) (Romani et al., 2021; Gerard et al., 2020; Falcao et al., 2021; Darko et al., 2020; Skalafouris et al., 2021; Cocoros et al., 2021; Simpson et al., 2020; de Lusignan et al., 2020; Burke et al., 2021; Gale et al., 2021; Mitchell et al., 2021; Knight et al., 2020) or by WHO (Chouchana et al., 2021; Gerard et al., 2021; WHO, 2019) and managed by various entities, such as universities (Simpson et al., 2020; de Lusignan et al., 2020; Burke et al., 2021; Gale et al., 2021; Knight et al., 2020), national pharmacovigilance centers (Romani et al., 2021; Falcao et al., 2021; Darko et al., 2020), governmental organizations (Cocoros et al., 2021; Mitchell et al., 2021), WHO (Chouchana et al., 2021; WHO, 2019), a monitoring center (Chouchana et al., 2021), or a hospital (Skalafouris et al., 2021). Six systems were fully supported by national governments (Cocoros et al., 2021; Simpson et al., 2020; Burke et al., 2021; Gale et al., 2021; Mitchell et al., 2021; Knight et al., 2020), two by WHO (Chouchana et al., 2021; WHO, 2019), and one partially by the government with grants and commercial investment (de Lusignan et al., 2020). The funding sources for the remaining four systems were not disclosed; however, three were managed by national pharmacovigilance centers or similar organizations (Romani et al., 2021; Falcao et al., 2021; Darko et al., 2020), and one was applied in a hospital (Skalafouris et al., 2021). Ten systems had extensive partners (Chouchana et al., 2021; Romani et al., 2021; Falcao et al., 2021; Darko et al., 2020; WHO, 2019; Cocoros et al., 2021; de Lusignan et al., 2020; Gale et al., 2021; Mitchell et al., 2021; Knight et al., 2020), including six with related international connections (Chouchana et al., 2021; Romani et al., 2021; Falcao et al., 2021; Darko et al., 2020; WHO, 2019; Cocoros et al., 2021), whereas the remaining three did not provide sufficient information (Skalafouris et al., 2021; Simpson et al., 2020; Burke et al., 2021) (Supplementary 1).

### 3.2 Specific characteristics of active surveillance systems

Among the 13 AS systems, six focused on safety (Chouchana et al., 2021; Gerard et al., 2021; Romani et al., 2021; Gerard et al., 2020; Falcao et al., 2021; Darko et al., 2020; Skalafouris et al., 2021; WHO, 2019), one on effectiveness (Burke et al., 2021), three on both (Cocoros et al., 2021; Simpson et al., 2020; de Lusignan et al., 2020), and three provided descriptive treatment data (Gale et al., 2021; Mitchell et al., 2021; Knight et al., 2020). Five safety systems were in collaboration with national pharmacovigilance centers (Chouchana et al., 2021; Gerard et al., 2021; Romani et al., 2021; Gerard et al., 2020; Falcao et al., 2021; Darko et al., 2020; WHO, 2019), two were WHO programs (Chouchana et al., 2021; Gerard et al., 2021; WHO, 2019), three were national systems connected to EudraVigilance or WHO’s VigiBase (Chouchana et al., 2021; Gerard et al., 2021; Romani et al., 2021; Gerard et al., 2020; Falcao et al., 2021), and one is a PharmaCheck system in a university hospital (Skalafouris et al., 2021). The FDA’s sentinel system (Cocoros et al., 2021), Scottish Early Pandemic Evaluation and Enhanced Surveillance of COVID-19 (EAVE II) (Simpson et al., 2020), and Oxford Royal College of General Practitioners Clinical Informatics digital hub (ORCHID) (de Lusignan et al., 2020) examined the safety and effectiveness of COVID-19 treatment, whereas the latter two were in the protocol stage. There is one effectiveness system, the REACT COVID-19 platform (Burke et al., 2021), that is ongoing, in which COVID-19-related complications have also been encountered. The three national AS systems (Gale et al., 2021; Mitchell et al., 2021; Knight et al., 2020) on COVID-19 epidemiology and clinical manifestations described drug treatments for ongoing or future intervention studies and did not directly monitor drug safety or effectiveness. Overall, the 13 AS systems enabled rapid response to COVID-19 by detecting early signals, quantifying adverse event incidences, improving monitoring efficiency, and collecting data for informed treatments and further clinical research (Supplementary 2).

### 3.3 Specific capabilities of active surveillance systems

The data sources, collection, submission, processing, accessing, and timeliness of the AS systems are outlined in Supplementary 3 and simplified in Table 1. Data sources for these systems were databases (Chouchana et al., 2021; Gerard et al., 2021; Romani et al., 2021; Gerard et al., 2020; Darko et al., 2020; Simpson et al., 2020), electronic medical records (EMRs) including chart review and clinical notes (Falcao et al., 2021; Skalafouris et al., 2021; WHO, 2019; Cocoros et al., 2021; de Lusignan et al., 2020; Burke et al., 2021), adverse drug reaction reports (Romani et al., 2021; Gerard et al., 2020; Darko et al., 2020), and questionnaires or report cards (WHO, 2019; Gale et al., 2021; Mitchell et al., 2021; Knight et al., 2020). Health professionals played a crucial role in data collection, along with other contributors such as pharmaceutical companies, patients, and individuals who were encouraged to report adverse events (Chouchana et al., 2021; Gerard et al., 2021; Falcao et al., 2021; WHO, 2019; Cocoros et al., 2021). Various tools were designed to enhance data collection efficiency, such as individual case safety reports (ICSRs) (Chouchana et al., 2021; Gerard et al., 2021; Darko et al., 2020), standardized data forms (Mitchell et al., 2021), and master protocols with a code list for data elements (Cocoros et al., 2021; de Lusignan et al., 2020). The WHO CEM study collected data through various channels, including a mobile application, website, paper diary, health facility visits, medical notes, and study staff interactions via telephone calls or home visits (WHO, 2019). The REACT-COVID-19 platform continuously updated longitudinal patient data during hospital stays and 12 months post-discharge from acute admission (Burke et al., 2021).

Data were submitted to surveillance organizations such as WHO VigiBase (Chouchana et al., 2021; Gerard et al., 2021), national pharmacovigilance centers (Romani et al., 2021; Gerard et al., 2020; Falcao et al., 2021; Darko et al., 2020), and the Public Health Agency of Canada (Mitchell et al., 2021), with secure methods (WHO, 2019; de Lusignan et al., 2020; Gale et al., 2021; Mitchell et al., 2021). The FDA’s sentinel system employed a Common Data Model to run identical computer programs across locations (Cocoros et al., 2021). Data processing involves checking, cleaning, and verification by epidemiologists or clinical informatics groups to solve missing data or data inconsistencies (Romani et al., 2021; Gerard et al., 2020; de Lusignan et al., 2020; Mitchell et al., 2021). Then, data were analyzed by investigator teams for early signals, prescriber alerts, and causality assessment (Chouchana et al., 2021; Gerard et al., 2021; Romani et al., 2021; Gerard et al., 2020; Falcao et al., 2021; Darko et al., 2020; Skalafouris et al., 2021; WHO, 2019; Gale et al., 2021). Data were pseudonymized, coded, and linked to other data sources or were electronically archived and retained (Darko et al., 2020; WHO, 2019; Cocoros et al., 2021; de Lusignan et al., 2020; Burke et al., 2021; Gale et al., 2021; Knight et al., 2020). Special data, such as ECGs, underwent interdisciplinary reviews (Romani et al., 2021; Gerard et al., 2020).

The REACT platform (Burke et al., 2021), VigiBase (Chouchana et al., 2021; Gerard et al., 2021), FDA’s sentinel system (Cocoros et al., 2021), and ORCHID Hub (de Lusignan et al., 2020) provide access to team members, pharmacovigilance centers, partners, or membership, respectively. In the WHO CEM study, the designated data manager was provided with a password-protected online database for data analysis (WHO, 2019). The WHO tools VigiLyze and VigiAccess enable national centers and the public to access VigiBase data (Gerard et al., 2021; Romani et al., 2021). The ORCHID system uses common data models to facilitate international studies (de Lusignan et al., 2020). Data-sharing was considered in the Canadian Nosocomial Infection Surveillance Program (CNISP) (Mitchell et al., 2021) and accepted in the United Kingdom Obstetric Surveillance System (UKOSS) (Knight et al., 2020). Meta-data were generated in EAVE II (Simpson et al., 2020), and aggregate reports were obtained in CNISP (Mitchell et al., 2021). Program websites, publications, and social media were used to disseminate the regular analysis reports to researchers, healthcare practitioners, policymakers, market authorization holders, and patients (Chouchana et al., 2021; Gerard et al., 2021; Romani et al., 2021; Gerard et al., 2020; Falcao et al., 2021; Darko et al., 2020; Skalafouris et al., 2021; WHO, 2019; Cocoros et al., 2021; Simpson et al., 2020; de Lusignan et al., 2020; Burke et al., 2021; Gale et al., 2021; Mitchell et al., 2021; Knight et al., 2020). Near real-time data use was implemented in the FDA’s Sentinel System (Cocoros et al., 2021), the ORCHID Hub (de Lusignan et al., 2020), the REACT platform (Burke et al., 2021), and the CNISP program (Mitchell et al., 2021). Reports of severe adverse events (SAEs) were submitted in real time in the WHO CEM study (WHO, 2019), while the Portugal national pharmacovigilance system (NPS) provided updates as soon as possible (Falcao et al., 2021). In nine systems, data were updated daily, weekly, every 2 weeks, or continuously (Chouchana et al., 2021; Gerard et al., 2021; Darko et al., 2020; Skalafouris et al., 2021; Cocoros et al., 2021; Simpson et al., 2020; de Lusignan et al., 2020; Burke et al., 2021; Gale et al., 2021; Knight et al., 2020). Meanwhile, the timely analysis produced weekly, monthly, and quarterly reports, guidance, recommendations, research findings, and developments in 11 systems (Chouchana et al., 2021; Gerard et al., 2021; Romani et al., 2021; Gerard et al., 2020; Darko et al., 2020; Skalafouris et al., 2021; WHO, 2019; Gale et al., 2021; Mitchell et al., 2021; Knight et al., 2020).

### 3.4 Applications of the 13 active surveillance systems

The WHO Programme for International Drug Monitoring (PIDM) was identified in two similar pharmacovigilance analyses (Chouchana et al., 2021; Gerard et al., 2021), while the French pharmacovigilance network was identified in two others (Romani et al., 2021; Gerard et al., 2020). One of the two included analyses was chosen as an example for applying each of these two systems (Chouchana et al., 2021; Romani et al., 2021). The key components of 13 studies utilizing the 13 AS systems for COVID-19 treatment are presented in Supplementary 4 and simplified in Table 1. Nine of the 13 studies were prospective observational studies (mainly cohort studies) for either safety, effectiveness, or both (Falcao et al., 2021; Darko et al., 2020; Skalafouris et al., 2021; WHO, 2019; Simpson et al., 2020; Burke et al., 2021; Gale et al., 2021; Mitchell et al., 2021; Knight et al., 2020), two were retrospective studies for pharmacovigilance analyses (Chouchana et al., 2021; Romani et al., 2021), one reported as a surveillance report (Cocoros et al., 2021), and one as a surveillance platform (de Lusignan et al., 2020). The goals were to provide adverse drug reaction (ADR) and SAE analyses in six safety surveillance studies, assess the effectiveness of therapeutic interventions in four studies (Cocoros et al., 2021; Simpson et al., 2020; de Lusignan et al., 2020; Burke et al., 2021), and describe drug treatment in three national surveillance studies (Gale et al., 2021; Mitchell et al., 2021; Knight et al., 2020).

Eight studies reported results from January to August 2020 (Chouchana et al., 2021; Romani et al., 2021; Falcao et al., 2021; Darko et al., 2020; Skalafouris et al., 2021; Gale et al., 2021; Mitchell et al., 2021; Knight et al., 2020), four were ongoing (Burke et al., 2021; Gale et al., 2021; Mitchell et al., 2021; Knight et al., 2020), and three were at the protocol stage (WHO, 2019; Simpson et al., 2020; de Lusignan et al., 2020). All studies focused on COVID-19 patients: seven involved hospitalized patients (Falcao et al., 2021; Skalafouris et al., 2021; Cocoros et al., 2021; Burke et al., 2021; Gale et al., 2021; Mitchell et al., 2021; Knight et al., 2020), four included pregnant women and children (Cocoros et al., 2021; Gale et al., 2021; Mitchell et al., 2021; Knight et al., 2020), and two were conducted in low- and middle-income countries (Darko et al., 2020; WHO, 2019).

Treatments used for COVID-19 included the following: remdesivir (REM) (Chouchana et al., 2021; Falcao et al., 2021; Cocoros et al., 2021; Knight et al., 2020), lopinavir/ritonavir (LPVr) (Darko et al., 2020; Skalafouris et al., 2021; Knight et al., 2020), oseltamivir (Mitchell et al., 2021; Knight et al., 2020), molnupiravir (WHO, 2019), and other antivirals (Burke et al., 2021; Gale et al., 2021; Mitchell et al., 2021; Knight et al., 2020); azithromycin (AZI) (Darko et al., 2020; Mitchell et al., 2021), doxycycline (Darko et al., 2020), and other antibiotics (Simpson et al., 2020; Burke et al., 2021; Gale et al., 2021; Mitchell et al., 2021); hydroxychloroquine (HCQ) (Romani et al., 2021; Falcao et al., 2021; Darko et al., 2020; Skalafouris et al., 2021; Mitchell et al., 2021) and chloroquine (CQ) (Darko et al., 2020); corticosteroids (Burke et al., 2021; Gale et al., 2021; Mitchell et al., 2021; Knight et al., 2020), including methylprednisolone (Darko et al., 2020) and dexamethasone (Cocoros et al., 2021); monoclonal antibodies (Cocoros et al., 2021), immunoglobulin (Gale et al., 2021), heparin (Cocoros et al., 2021), low-molecular-weight heparin (Cocoros et al., 2021), and anti-arrhythmic treatment (Gale et al., 2021); existing or newly developed therapies (Simpson et al., 2020); and concurrent medication influencing COVID-19 outcomes (e.g., ACE inhibitors/ARBs) (de Lusignan et al., 2020; Burke et al., 2021).

The included outcomes were as follows: ADR incidence (Falcao et al., 2021), ADRs and serious ADRs (Chouchana et al., 2021; Romani et al., 2021; Darko et al., 2020; WHO, 2019), drug discontinuation (Chouchana et al., 2021; Falcao et al., 2021), causality assessment (Falcao et al., 2021; Darko et al., 2020), and consumption of antibiotics (de Lusignan et al., 2020); clinical outcomes including death (Cocoros et al., 2021; de Lusignan et al., 2020; Burke et al., 2021; Gale et al., 2021; Mitchell et al., 2021; Knight et al., 2020), mechanical ventilation or extracorporeal membrane oxygenation (ECMO) (Cocoros et al., 2021; de Lusignan et al., 2020; Burke et al., 2021; Mitchell et al., 2021), hospital admission (de Lusignan et al., 2020; Knight et al., 2020) or length of stay (Gale et al., 2021), intensive care unit (ICU) admission (de Lusignan et al., 2020; Mitchell et al., 2021; Knight et al., 2020) or length of stay (Cocoros et al., 2021), discharge status (Cocoros et al., 2021; Burke et al., 2021; Gale et al., 2021; Mitchell et al., 2021; Knight et al., 2020), medication changes during clinical follow-up for COVID-19 complications (Burke et al., 2021), maternal and perinatal outcomes (WHO, 2019; Knight et al., 2020), and other COVID-19-related outcomes (Skalafouris et al., 2021; Simpson et al., 2020; de Lusignan et al., 2020; Burke et al., 2021).

### 3.5 Evaluation of 13 active surveillance systems

No formal assessment of the reliability and validity of the 13 AS systems was found. However, various methods were used to improve the quality of surveillance data and reports. VigiBase utilized predefined quality criteria to check each coming report, applied the vigiMatch method to detect duplicates, and coded indications for suspected drugs using COVID-19-related terms in MedDRA (Chouchana et al., 2021; Gerard et al., 2021). The WHO CEM study provided training for all staff and conducted automatic quality checks for data anomalies (WHO, 2019). The FDA’s sentinel system approved data for analysis following thorough quality assurance checks. Additionally, most sentinel data partners can access full-text medical records to validate their electronic data or provide clinical details (Cocoros et al., 2021). Other systems used regular validation checks on databases (Simpson et al., 2020), study audits (Gale et al., 2021), accurate coding in clinical practice (de Lusignan et al., 2020), double-entered data (Gale et al., 2021), and adding historical comparison cohorts (Romani et al., 2021; Knight et al., 2020) (Supplementary 5).

Four systems demonstrated notable strengths: over 90% completeness and accuracy of Scottish Morbidity Record datasets in the EAVE II (Simpson et al., 2020), high reporting levels by United Kingdom pediatricians in British Paediatric Surveillance Unit (BPSU) surveillance (Gale et al., 2021), rapid and accurate data collection in a public health emergency in UKOSS (Knight et al., 2020), and more fluid longitudinal data capture in the REACT COVID-19 platform (Burke et al., 2021). However, some limitations were observed, such as underreporting of ADRs (Chouchana et al., 2021; Romani et al., 2021; Falcao et al., 2021; Darko et al., 2020), residual confounders in retrospective design (Chouchana et al., 2021), selection bias in prospective observational studies (WHO, 2019), descriptive nature of analyses without drawing causal inferences (Mitchell et al., 2021), incomplete datasets following the longitudinal clinical care path (Burke et al., 2021), small patient numbers (Darko et al., 2020), and short follow-up periods (WHO, 2019). Regarding privacy and data security, eight systems ensured compliance with applicable data protection and privacy laws, affirming that data handling adhered to security measures and unauthorized access was prevented (Supplementary 5) (Chouchana et al., 2021; WHO, 2019; Cocoros et al., 2021; de Lusignan et al., 2020; Burke et al., 2021; Gale et al., 2021; Mitchell et al., 2021; Knight et al., 2020).

## 4 Discussion

The COVID-19 pandemic highlighted the need for rapid and extensive surveillance systems to monitor the safety and effectiveness of therapeutic interventions and to support decision-making under conditions of uncertainty. In response to this urgency, in this scoping review, we identified and mapped 13 AS systems implemented during the pandemic to evaluate health products, particularly COVID-19 treatments. Together, these systems illustrate how proactive, system-embedded use of routinely collected health data enabled earlier detection of safety signals, timely assessment of real-world effectiveness, and more responsive clinical and regulatory action than passive surveillance alone. These findings position AS as a critical component of modern pharmacovigilance and learning health systems and underscore the importance of sustained, well-designed surveillance infrastructures to support patient care, healthcare system resilience, and preparedness for future public health emergencies. However, only one of these systems was explicitly designed to follow up on COVID-19 complications and related medication changes for a 12-month period (Burke et al., 2021). This system provides a unique contribution by delving into the longer-term implications of COVID-19 treatment, shedding light on potential complications and necessary adjustments in medication regimens over an extended period. Notably, 11 of the 13 systems had previously been established for other diseases, allowing for their immediate application to COVID-19 in very early pandemic, a sign of their adaptability and versatility.

The early phase of the pandemic witnessed a quick response, with existing AS systems quickly re-used mainly to monitor drug safety in COVID-19. These systems were instrumental in providing crucial surveillance results for the first few months of 2020, particularly insights into the safety profile of drugs used in COVID-19 treatment. It was noted that the systems focused on effectiveness were at an initial stage, either under protocol or ongoing.

As the pandemic progressed, a shift was aimed at developing new surveillance studies, exemplified by the WHO multicenter multi-country prospective cohort study for the safety of molnupiravir, a new approved drug, in 2022 (WHO, 2019). Furthermore, active surveillance was extended for a more prolonged follow-up of COVID-19 treatment, as demonstrated by the UKOSS, which continued its surveillance of pregnant women participating in clinical trials (NPEU, 2025). Some systems, such as the CNISP, transitioned into year-round seasonal surveillance for COVID-19 and influenza in subsequent years (IPAC, 2021). The increasing digitization of medical records and health information has significantly influenced recent surveillance efforts, with tools such as the FDA Sentinel Initiative leveraging claims or electronic health record data for AS (Dhodapkar et al., 2022). This shift toward electronic records, large-scale databases, computerized techniques, and online tools has significantly enhanced the efficiency of active surveillance and then facilitated near-real-time data access.

The scoping review encompasses three population-based national AS systems: the BPSU, CNISP, and UKOSS. Although they were primarily repurposed to monitor the spread of COVID-19, they also provided valuable information on drug treatment for COVID-19, with nationally involving children, pregnant women, and hospitalized patients. Despite their limit to descriptive treatment data, these systems emerged as efficient tools for monitoring COVID-19 incidents, risk factors, and clinical manifestations, offering a real-world clinical context and robust data to help inform ongoing or future intervention studies.

Although many AS systems deployed during the COVID-19 pandemic were limited by heterogeneity in structure, reporting, and transparency, it is important to situate these systems within a broader ecosystem of adaptive evidence-generation approaches that matured during the same period. In this review, all study designs were eligible if they reported AS methods and provided population-level active data access and reporting, including adaptive clinical trials that met these criteria. In particular, adaptive platform trials such as REMAP-CAP, RECOVERY, and PRINCIPLE demonstrated how preplanned and flexible infrastructures can generate rapid, high-impact evidence during public health emergencies (Harris and Adalja, 2023). These trials enabled the simultaneous evaluation of multiple interventions, applied prespecified adaptive decision rules to add or discontinue treatment arms, and disseminated clinically actionable findings in near-real time, directly informing guideline updates and frontline care (Harris and Adalja, 2023).

Although adaptive platform trials were not designed primarily as post-authorization safety surveillance systems, they addressed several structural limitations commonly observed in traditional AS approaches, including delays in feedback and fragmented reporting. Lessons from these platforms, such as centralized governance, standardized data capture, continuous interim analyses, and rapid dissemination mechanisms, are highly relevant to the future design of AS systems for both safety and effectiveness. Together, AS and adaptive platform trials function as complementary components of learning health systems, linking real-world signal detection with rapid confirmatory evaluation to accelerate the translation of evidence into clinical practice during both emergency and routine care contexts.

## 5 Implications for future pandemics and emerging health threats

The rapid adaptation of AS systems during the COVID-19 pandemic demonstrates their potential as foundational infrastructure for future public health emergencies. Many of the AS systems identified in this review, including national pharmacovigilance networks, multisite data platforms, and hospital electronic medical record-linked systems, already exhibit key preparedness features such as flexible system design, integrated data environments, and near-real-time analytic capacity. The use of common data models, secure multisite data sharing, and frequent data updates enables rapid redirection of these systems to monitor novel pathogens, new therapeutics, and emerging safety concerns. Sustaining and strengthening AS infrastructure through pre-positioned analytic tools, automated signal detection, and coordinated governance can support timely evidence generation and position AS as an integral component of learning health systems and global pandemic preparedness.

Ethical considerations related to data privacy, patient consent, and public trust are also critical as AS systems are expanded beyond COVID-19. Across many initiatives reviewed, privacy protections were embedded in system design through de-identification or pseudonymization, secure data environments, restricted access, and compliance with applicable data protection requirements. Although many AS systems rely on secondary use of routinely collected clinical or public health data, often under authorized consent waivers during public health emergencies, systems involving direct patient participation generally incorporated study-specific consent procedures. Embedding privacy-by-design principles, transparent governance, and clear communication about data use will be essential to maintaining public trust and ensuring the long-term sustainability of AS systems for monitoring safety and effectiveness during future pandemics and other emerging health threats.

## 6 Limitations

This scoping review has several important limitations that should be interpreted in the context of the rapidly evolving use of AS during the COVID-19 pandemic. Most notably, the lack of detailed information on how data were accessed, captured, processed, and reported represents a central limitation of the existing evidence base and should be interpreted in the context of the rapidly evolving use of AS during the COVID-19 pandemic. This lack of transparency made it difficult to determine with certainty whether the described systems met eligibility criteria for active surveillance. Using “active data access” as an eligibility requirement may, therefore, have excluded potentially relevant initiatives with inadequate or unclear reporting on data flows, governance structures, analytic pipelines, data update frequency, or timeliness of reporting.

In addition, the AS systems identified in this review were highly heterogeneous in scope, maturity, and intended use. They ranged from repurposed national pharmacovigilance networks and sentinel programs with established infrastructures and broad population coverage to hospital-based electronic medical record alert systems and cohort platforms designed for localized monitoring or specific outcomes. Systems also differed in their stage of development, analytic sophistication, and emphasis on safety monitoring, effectiveness assessment, or descriptive utilization patterns. This heterogeneity, combined with selective or high-level reporting, limited direct comparability across systems and precluded formal assessment of methodological rigor or performance. Importantly, these limitations reflect broader structural challenges in active surveillance reporting rather than weaknesses of the review itself. At the same time, the identification of gaps in reporting, transparency, and standardization represents a central contribution of this review. Overall, these findings highlight clear priorities for improved methodological standardization, more transparent documentation, and future development of active surveillance systems, reinforcing the relevance and value of this work for advancing the field.

## 7 Conclusion

The COVID-19 pandemic accelerated the development and repurposing of AS to address urgent challenges in evaluating the safety and effectiveness of therapeutic interventions. This scoping review maps the dynamic landscape of AS systems implemented during the pandemic and highlights their essential role in generating timely, real-world evidence when conventional data sources were limited or delayed. Beyond descriptive mapping, the findings demonstrate how AS supported earlier signal detection, informed clinical and regulatory decision-making, and contributed to healthcare system resilience during periods of uncertainty. This review also situates AS within a broader learning health system, in which adaptive platform trials operated alongside AS as complementary approaches, providing rapid confirmatory evidence that translated emerging signals into clinical guidance and informed future system design. At the same time, important gaps in transparency, standardization, and reporting were identified, indicating clear priorities for improving system documentation and methodological rigor. Strengthening integrated, ethically governed AS infrastructures, while maintaining data privacy, appropriate consent practices, and public trust, will be critical for future pandemic preparedness and for sustaining AS as a core component of learning health systems capable of delivering rapid, reliable evidence during public health emergencies.
