# Supplementary material for: Active surveillance for the safety and effectiveness of health products for COVID-19: a scoping review
Source: Front Pharmacol. 2026 Apr 2;17:1752571. doi: 10.3389/fphar.2026.1752571 (PMC13084158; doi:10.3389/fphar.2026.1752571)
Supplement: Supplementary file 1 [file DataSheet1.docx]

**Active Surveillance for safety and effectiveness of health products for COVID-19: A scoping review**

**Supplementary 1 - General characteristics of the thirteen active surveillance systems**

| **AS systems** | **Organizations managing AS systems** | **Partnership** | **Country** | **Funding** | **Year established** | **Year/ Month used for COVID-19** |
| --- | --- | --- | --- | --- | --- | --- |
| **Active surveillance on safety*** | | | | | | |
| WHO PIDM program [20,21] | Uppsala Monitoring Center (UMC), on behalf of WHO | Member countries of the WHO PIDM | International | WHO | 1968 | January 2020 |
| French pharmacovigilance network [22,23] | National Agency, Safety of Medicines and Health Products | European notification database (EudraVigilance),  WHO database Vigibase | France | No funding | 1985 | March 2020 |
| NPS system [24] | The Portuguese National Authority for Medicines and Health Products (INFARMED) | EudraVigilance | Portugal | NR | 1992 | March 2020 |
| SafetyWatch system [25] | National Pharmacovigilance Centre | WHO PIDM; The Heads of the Treatment Centres, COVID-19 Case Management Teams and the Institutional Contact Persons | Ghana | NR | 2001 | April 2020 |
| PharmaCheck system [26] | Geneva University Hospital | NR | Switzerland | NR | 2020 | February 2020 |
| CEM method [27] | WHO | National Ethics Review Committee in different countries | International | WHO | Before 2020 | March 2022 |
| **Active surveillance on safety and effectiveness*** | | | | | | |
| FDA's sentinel system [28] | FDA-US | Sentinel partnerships: electronic health record(s)-based organizations and networks— Hospital Corporation of America (HCA) Healthcare, the National Patient-Centered Clinical Research Network (PCORnet), and several Electronic Health Record (EHR) data aggregators (TriNetX, Veradigm, IBM Explorys); international collaboration with the European Medications Agency (EMA) | US | FDA | 2009 | April 2020 |
| EAVE II surveillance [29] | University of Edinburgh and Public Health Scotland | Albasoft Ltd, the trusted third party | Scotland | Medical Research Council and Scottish Government | 2015 (EAVE) | June 2020  (EAVE II) |
| ORCHID digital hub [30] | University of Oxford, Royal College of General Practitioners Research and Surveillance Centre (RCGP RSC) | Public Health England (PHE), NHS Digital and NHSX, FDA’s Sentinel, Observational Medical Outcomes Partnership | UK | RCGP RSC is principally funded by PHE. Grants and commercial investment to maintain the ORCHID | 1967  (RCGP RSC) | 2020 |
| **Active surveillance on effectiveness*** | | | | | | |
| REACT COVID-19 platform [31] | University of Southampton, University Hospital Southampton NHS Foundation Trust (UHSFT), and the digital Experimental Cancer Medicine Team | NR | UK | National Institute for Health Research (NIHR) | 2020 | March 2020 |
| **Active surveillance with descriptive treatment data*** | | | | | | |
| BPSU system [32] | Royal College of Paediatrics and Child Health, PHE, University College London Institute of Child Health [35] | NIHR Policy Research Unit in Maternal and Neonatal Health and Care, University of Oxford-National Perinatal Epidemiology Unit (NPEU) | UK | NIHR Policy Research Programme | 1985 | April 2020 |
| CNISP program [33] | Government of Canada | Public Health Agency of Canada, National Microbiology Laboratory, Association of Medical Microbiology and Infectious Disease Canada, Sentinel hospitals | Canada | Public Health Agency of Canada | 1994 | March 2020 |
| UKOSS system [34] | NPEU, Oxford population health, University of Oxford | Royal College of Obstetricians and Gynaecologists, Royal College of Midwives, Faculty of Public Health, Health Protection Agency, Department of Health Patient Safety Research Programme | UK | NIHR-Policy Research Programme, NIHR- Health Technology Assessment Programme [36] | 2005 | March 2020 |

AS, Active surveillance; BPSU, British Paediatric Surveillance Unit; CEM, Cohort Event Monitoring; CNISP, Canadian Nosocomial Infection Surveillance Program; EAVE, Early Estimation of Vaccine and Anti-Viral Effectiveness; EAVE II, Early Pandemic Evaluation and Enhanced Surveillance of COVID-19; EudraVigilance, European notification database; FDA, Food and Drug Administration; NHS, National Health Service; NHSX, NHS "user experience”; NIHR, National Institute for Health Research; NPEU, National Perinatal Epidemiology Unit; NPS, National pharmacovigilance system; NR, not reported; PHE, Public Health England; ORCHID, Oxford Royal College of General Practitioners Clinical Informatics Digital Hub; PIDM, Programme for International Drug Monitoring; RCGP RSC, Oxford Royal College of General Practitioners Research and Surveillance Centre; REACT, Research Evaluation Alongside Clinical Treatment; UK, United Kingdom; UKOSS, UK Obstetric Surveillance System; UHSFT, University Hospital Southampton NHS Foundation Trust; US, United States; US, United States; Vigibase, WHO global database of individual case safety reports; WHO, World Health Organization

*The categories were based on the information on overall introduction of active surveillance systems identified under the design in **Appendix 2**.

**Supplementary 2 - Specific characteristics of the thirteen active surveillance systems**

| **AS systems** | **Design (objectives, structure, methods, and size/scalability)** | **Adding value to existing surveillance capacities** |
| --- | --- | --- |
| **Active surveillance on safety** | | |
| WHO PIDM program [20,21] | WHO PIDM aims to swiftly identify suspected safety issues in medicinal products through international collaboration [37, 38]. VigiBase, the WHO's global database, compiles reported potential side effects from around 150 member countries, covering nearly 99% of the world's population. It's regularly updated and facilitates disproportionality analyses, serving as a central tool for UMC's signal detection and research. Timely recording and reporting of adverse events are imperative for gathering comprehensive data on the adverse drug reactions of COVID-19 treatments. | NR |
| French pharmacovigilance network [22,23] | In France, health professionals report adverse drug reactions (ADRs), especially if serious or unexpected, via regional Centers of Pharmacovigilance (RCPVs). These reports are centralized by the French Pharmacovigilance Database (FPVD), managed by a national network of 31 RCPVs. This network evaluates the real-world implications of expected and unexpected risks associated with drug treatments. The pharmacovigilance department utilizes EudraVigilance and the WHO's Vigibase databases. Mandated by the National Agency for the Safety of Medicines and Health Products on March 27, 2020, the RCPV of Nice investigated all reports of cardiotoxicity associated with hydroxychloroquine (HCQ), chloroquine (CQ), azithromycin (AZI), or lopinavir used empirically against COVID-19, and assesses cases of cardiotoxicity linked to HCQ use against COVID-19. | The profile of cardiac adverse drug reactions (CADRs) associated with HCQ differed significantly in COVID-19 patients compared to before. Cardiac monitoring facilitated the early detection of HCQ CADRs in COVID-19 patients. |
| NPS system [24] | The Portuguese NPS oversees the safety of medicines authorized for the national market, identifying and addressing ADRs and implementing safety measures as needed. Cases are then forwarded to EudraVigilance, managed by the European Medicines Agency, for continuous monitoring and in-depth analyses to detect safety signals.  This study evaluates suspected ADRs associated with REM and HCQ regimens in COVID-19 patients, reported to the Portuguese NPS over six months, using a prospective observational approach. | The study methodology, employing an active monitoring model, assessed both off-label prescriptions used at the pandemic's onset and the single authorized drug for COVID-19. This approach enabled the quantification of ADR incidence. |
| SafetyWatch system [25] | Ghana became a member of the WHO PIDM in 2001. The National Pharmacovigilance Centre developed a Safety Monitoring Plan for COVID-19 treatments and operates a system to monitor safety (SafetyWatch). The plan aims to ensure healthcare professionals report suspected ADRs related to Emergency Use Authorization medicines and other COVID-19 treatments. Data was collected from 40 treatment centers across Ghana's 16 regions. | The ADRs are closely monitored for potential signals as the medications remain in use for COVID-19. |
| PharmaCheck system [26] | PharmaCheck, integrated into the hospital information system at Geneva University Hospitals in February 2020, screens patients in internal medicine wards daily. Pharmacists utilize the electronic screening tool to identify 20 high-risk situations, that could lead to adverse drug events (ADEs), especially those related to LPVr and HCQ prescriptions that deviate from institutional guidelines or have contraindications. | PharmaCheck now conducts centralized pharmaceutical screening, replacing traditional reviews due to the COVID-19 patient surge. Its scope includes monitoring LPVr and HCQ prescriptions. |
| CEM method [27] | The cohort event monitoring (CEM) method is designed to detect safety signals in a master protocol for a multinational observational study by WHO. This prospective single-arm cohort study aims to actively monitor the safety of molnupiravir for non-severe COVID-19 treatment. As an AS method, CEM is used to follow-up early molnupiravir treatment to promptly collect post-market safety data, surpassing the limitations of RCTs by reflecting real-world clinical practice. The study actively monitors participants, including women and children exposed in pregnancy or breastfeeding, with a target enrollment of 30,000 patients across low-to-middle income countries, representing all six WHO regions. | NR |
| **Active surveillance on safety and effectiveness** | | |
| FDA's sentinel system [28] | The Sentinel system was created to enhance the national capacity for evaluating post-market medical product safety, expanded and upgraded for COVID-19 assessment in April 2020, and enabled real-time monitoring of the virus using routinely collected electronic health data. This comprehensive approach examines the usage, safety, and potentially effectiveness of medical products for COVID-19 prevention and treatment. Sentinel's core Data Partners include representation from all US 50 states. | Sentinel system actively monitors the treatments and outcomes of COVID-19 patients using near real-time claim data and novel methods, enhancing its ability to respond quickly. The data model, sources, and database were improved. |
| EAVE II surveillance [29] | EAVE II was established to enhance a national pandemic reporting platform. It utilizes anonymized patient data to investigate COVID-19 epidemiology and assess the effectiveness and safety of new or existing therapies. Data collection aims to cover over 91% of the Scottish population, including all residents registered with general practice. | EAVE II repurposed and expanded the hibernated EAVE project into a proven platform for studies on COVID-19 and antiviral assessment under the NIHR Pandemic Preparedness Research Portfolio. |
| ORCHID digital hub [30] | ORCHID is a new integrated digital health hub established to extract routinely collected general practice electronic health data to complement the recording of adverse events and key outcomes for clinical trials and enhanced communicable disease surveillance, including COVID-19. Leveraging the existing Oxford RCGP RSC infrastructure and practice relationships, ORCHID minimizes large-volume data extracts and reduced trial workload. All general practices in England with a supported primary care computerized medical record system are eligible to participate through the Oxford RCGP RSC network. | The COVID-19 pandemic led to a surge in new practices joining the Oxford RCGP RSC network to support national surveillance. |
| **Active surveillance on effectiveness** | | |
| REACT COVID-19 platform [31] | The REACT platform assesses early clinical benefits of new cancer medicines in early-phase drug trials and facilitates rapid upload and interpretation of clinical data during the COVID-19 pandemic. The objective is to establish a real-time database of well-characterized patients with COVID-19 from prehospital to tertiary care and subsequent COVID-19 related complications from discharge to follow-up, and to evaluate the efficacy of current best practice management strategies in an 1100-bed tertiary hospital (UHSFT). | The longitudinal and automated clinical data collection via the REACT platform during the COVID-19 pandemic offers precise details to identify early disease severity markers and their impact on disease progression. The REACT database quickly identifies potential clinical trial participants and offers visual data interpretation for hypothesis generation. |
| **Active surveillance with descriptive treatment data** | | |
| BPSU system [32] | The BPSU platform conducts national studies on rare neonatal and pediatric diseases, utilizing the orange card system, which has set a standard for national surveillance in various specialties worldwide. This method ensures accurate estimation of regional and national incidence, clinical data, and outcomes, with over 90% response rate from pediatricians and neonatologists monthly. Since April 1, 2020, all consultant pediatricians in the UK have received a weekly electronic BPSU reporting card to notify eligible cases of neonatal SARS-CoV-2 infection. Subsequently, the research team sends a study-specific data collection form to the notifying doctor. | AS via national systems, with robust population-based case ascertainment, efficiently provides accurate population-level data on COVID-19 incidence, clinical characteristics, and outcomes. Information addressing COVID-19 severity and management strategies is key to inform treatment decisions in the context of randomized trials. [39] |
| CNISP program [33] | CNISP conducts active surveillance for healthcare-associated infections and antimicrobial-resistant organisms in Canadian acute care hospitals, aiming to prevent, control, and reduce these infections. Surveillance was expanded on March 15, 2020, to include all hospitalized patients with laboratory-confirmed COVID-19. Forty-eight CNISP hospitals across 9 provinces conducted prospective sentinel surveillance of adult and pediatric patients with COVID-19. | This program enhances understanding of COVID-19 epidemiology and clinical manifestations in Canadian acute care hospitals for both adults and children. |
| UKOSS system [34] | The UKOSS system collects data on severe maternal morbidity from all consultant-led maternity units in the UK through a monthly case-collection scheme. A national prospective observational cohort study using UKOSS was designed in 2012 and activated as an urgent public health study in response to the SARS-CoV-2 pandemic. Clinicians reported all pregnant women with confirmed SARS-CoV-2 infection admitted to the hospital via a live reporting link. The UKOSS team then collected detailed information on risk factors, management, and outcomes for each case, determining the incidence of hospitalization with pandemic COVID-19 infection in pregnancy and assessing outcomes in pregnancy for mother and infant. | The national observational data collection method offers the best rapidly available high-quality evidence to inform clinical and public health policy and management guidance, considering the ethical and other challenges of conducting clinical trials in pregnant women. |

ADR, adverse drug reaction; AS, Active surveillance; BPSU, British Paediatric Surveillance Unit; CADRs, cardiac adverse drug reactions; CEM, Cohort Event Monitoring; CNISP, Canadian Nosocomial Infection Surveillance Program; EAVE, Early Estimation of Vaccine and Anti-Viral Effectiveness; EAVE II, Early Pandemic Evaluation and Enhanced Surveillance of COVID-19; EudraVigilance, European notification database; FDA, Food and Drug Administration; HCQ, hydroxychloroquine; LPVr, lopinavir/ritonavir; NIHR, National Institute for Health Research; NPS, National Pharmacovigilance System; NR, not reported; ORCHID, Oxford Royal College of General Practitioners Clinical Informatics Digital Hub; PIDM, Programme for International Drug Monitoring; RCGP RSC, Oxford Royal College of General Practitioners Research and Surveillance Centre; RCPV, regional Centers of Pharmacovigilance; REACT, Research Evaluation Alongside Clinical Treatment; REM, remdesivir; UHSFT, University Hospital Southampton NHS Foundation Trust; UK, United Kingdom; UKOSS, UK Obstetric Surveillance System; UMC, Uppsala Monitoring Center; US, United States; Vigibase, WHO global database of individual case safety reports; WHO, World Health Organization

**Supplementary 3 - Specific capabilities of the thirteen active surveillance systems**

| **AS systems** | **Type of data source** | **Data collection** | **Data** | | | **Timeliness** |
| --- | --- | --- | --- | --- | --- | --- |
|  |  |  | **Submission** | **Processing** | **Accessing** |  |
| **Active surveillance on safety** | | | | | | |
| WHO PIDM program [20,21] | VigiBase and Individual Case Safety Reports (ICSRs) | Health professionals, patients, and pharmaceutical companies report suspected adverse drug reactions (ADRs) to national pharmacovigilance centers. ICSRs, also known as voluntary reports, are generated during the post-marketing phase of drugs. ICSRs include patient data such as age, sex, suspected and concomitant drugs, ADRs, date of occurrence, and seriousness. | Member countries of the WHO PIDM send the reports to VigiBase. | ICSRs are analyzed locally and could result in regulatory action within the respective country. At UMC, clinical assessment occurs exclusively for combinations of drug and adverse effect deemed highly likely to indicate a potential signal. | National and regional pharmacovigilance centers have access to VigiBase via VigiLyze for signal detection and strengthening. The WHO tool VigiAccess offers limited public access to some VigiBase data. | Most national centers provide quarterly reports or even more frequently. VigiBase is continuously updated, sometimes daily. |
| French pharmacovigilance network [22,23] | French Pharmacovigilance database,and ADR reports | The French Pharmacovigilance Network directed cardiac adverse drug reactions related to off-label use of HCQ, AZI, and LPVr in COVID-19 to the Nice Regional Center of Pharmacovigilance. Data collected included source of reporting, age, sex, therapeutic indication, CADRs, onset time, severity, narrative, diagnosis, and outcomes. ECGs were collected to confirm pathological trace and measure the QT interval. | The identified cases were transmitted to the Reginal center of Pharmacoviglance (RCPV) of Nice and individually screened to identify CADRs. | Pharmacovigilants reviewed each ADR case and requested missing data to initial health professionals. Trained residents examined ECGs under cardiologist supervision. Each Regional Center of Pharmacovigilance assessed the causality of drugs. | Pharmacovigilance databases are utilized for signal detection or replication, eventually confirming findings from prospective trials or preclinical studies. Institutional cases undergo systematic analysis in pharmacology and ADR interpretation. The FPVD-permits adding information regarding the clinical context, including pregnancy, misuse, medication error, overdose, etc. | Cardiac monitoring of COVID-19 patients allowed early detection of HCQ CADRs. |
| NPS system [24] | Patients’ Chart review and ADR reports | Health professionals and citizens can report suspected adverse reactions. A chart review was conducted for patients meeting inclusion criteria to determine ADR occurrences based on the institution's active monitoring model. A team of reviewers prospectively evaluated medical records for relevance. | Any suspected ADRs were reported to the Portuguese Pharmacovigilance System online. | The NPS team assessed drug-ADR causality. | The action triggered by the ADR was evaluated. | ADR report was sent to INFARMED as soon as possible. |
| SafetyWatch system [25] | Ghanaian pharmacovigilance database and ADR reports | The ICSRs included information such as the generic names of suspected and concomitant medications (including herbal products), patient age and sex, ADR description, seriousness criteria, causality assessment, suspected drug administration date, ADR onset and stop dates, and outcomes. | Healthcare professionals sent the ICSRs via a stimulated spontaneous reporting system. | The Ghanaian pharmacovigilance database stored ICSRs for medicines, and all reactions were coded using the Preferred Terms (PTs). Institutional Contact Persons received daily follow-ups and reminders on the need to follow-up and submit weekly updates to the National Pharmacovigilance Centre. | The Food and Drugs Authority's Technical Advisory Committee on Safety of Medicines assessed the causality of received ADR reports using the WHO–UMC system for standardized case causality assessment. The expectedness of each ADR was assessed using the Summary of Product Characteristics. | Daily follow-ups and weekly updates |
| PharmaCheck system [26] | Hospital’s EMR | PharmaCheck screens electronic health records in near real-time by aggregating information from the hospital's data lake, including drug prescriptions, laboratory values, vital signs, and medical problems. | NR | The pharmacist analyzes detected situations and alerts the prescriber if a risk of ADE is clinically relevant, recommending treatment adjustments. Four pharmacists rotated using the tool and followed up on situations requiring closer monitoring. | The pharmacists measured the distribution of alerts and triggers, the positive predictive value of each trigger, the proportion of alerts resulting in a recommendation for treatment adjustment or additional monitoring, and prescribers' acceptance of the recommendations. | PharmaCheck ran twice daily |
| CEM method [27] | Questionnaire and  medical records | All participants will complete questionnaires via a mobile app/website/paper diary/health facility visit/medical note or via study staff following telephone calls or home visits. Study staff administer and collect participants' baseline information (demographic and medical) and pre-exposure event data, along with covariates of interest when molnupiravir treatment starts. They will also gather information on concomitant medications, traditional medicine and supplements, non-adherence to treatment, and progression to severe COVID-19 disease. | Study staff/ patients will input data electronically at several time points. The data will be transferred and stored in a password-protected database with anonymous study IDs for participants. A central hub will be established to standardize collected data, regardless of the tool used. | The key-coded collected data will be securely stored at the WHO headquarters in Geneva and electronically archived and retained for three years. Each country will have access to its national data and share anonymized data via standard WHO data sharing agreements. Individual causality assessments for all SAEs will be conducted by national committees, with WHO support for local data platform development. | A designated data manager will get a password-protected copy of the online database for analysis. Monthly interim analyses will be done by a team member. A safety signal review group will investigate emerging signals. Reports of SAEs will be sent to national authorities immediately. A full study report will be ready within 20 weeks after the database lock. Safety concerns will be informed to market authorization holders, and study results will be shared with national regulatory authorities by local study teams. | Interim analyses conducted monthly.  Reports of SAEs sent in real time.  Full study report completed within 20 weeks after the database lock. |
| **Active surveillance on safety and effectiveness** | | | | | | |
| FDA's sentinel system [28] | US-based EHRs and administrative claims | A natural history Master Protocol guides various COVID-19 studies using claims, EHR, or linked data. The protocol includes code lists for data elements and addresses special populations like pregnant women and children. The Common Data Model comprises demographic details, diagnoses, procedures, drug dispensing, lab results, vital signs, and care eligibility dates. | Each core Data Partner transforms their data into the Sentinel Common Data Model, enabling identical computer program execution across locations. MyStudies smartphone app collects patient-reported data to complement routine Sentinel data and has been adapted to address the COVID-19 pandemic. | Data undergoes review and quality assurance by each organization before analysis. Sentinel leverages pandemic preparation activities to monitor care patterns and clinical outcomes, utilizing surveillance reports on COVID-19 census and clinical characteristics in HCA Healthcare. This includes identifying medication administration and assessing complications during hospitalization. | Sentinel teams publicly share analysis details, including code lists and analytic programs, and maintain this commitment during the pandemic. Each partner accesses near real-time EHR data, covering prescriptions and medication administrations. | Using near real-time claims data and keeping updated every 2 weeks |
| EAVE II surveillance [29] | Scottish national linked databases | A national linked dataset comprising primary care, hospitalization, mortality, and laboratory data will be assembled. These aggregated data include individual-level data from general practices linked to secondary and laboratory datasets, along with data from COVID-19 Community Hubs and Assessment Centers, as well as data on prescribing and administering medicines for inpatients. | NR | NR | The Community Health Index (CHI) number serves as a mandatory and unique identifier for accessing metadata. This metadata will also be accessible to Health Data Research UK Gateway through the Health Data Research Hub for Respiratory Health. | Monitoring the daily/weekly progress of the COVID-19 epidemic, evaluating the effectiveness of therapeutic interventions, and undertaking timely analyses. |
| ORCHID digital hub [30] | Computerized medical record (CMR) | Pseudonymized patient data will be extracted from general practice CMR systems, including demographic and clinical event data coded with Systematized Nomenclature of Medicine (SNOMED) and medication data coded with the Dictionary of Medicines and Devices. RCGP RSC uses Apollo Data Management Services for data extractions. | Encrypted data will be securely transported to the protected hub. | Data will be cleaned and checked by Clinical Informatics group members, Pseudonymized data will link within the hub primary care data to other CMR data sources via patient's NHS number. | The hub will be discoverable via membership in Health Data Research UK and European Health Data & Evidence Network (EDHEN) metadata repositories. An online application system enables access to study-ready or custom data sets. Data will be shared using common data models for international studies. | Providing near-real-time data~~,~~ and weekly updates on the latest surveillance and research findings, developments within the hub, and tips for enhancing data quality. |
| **Active surveillance on effectiveness** | | | | | | |
| REACT COVID-19 platform [31] | Hospital’s EMR and clinical notes | Core demographic and clinical data is extracted from electronic hospital records. Additional COVID-19 clinical course data is manually extracted from clinical notes and uploaded to the database by clinical researchers where electronic export is impossible. Subsequent data capture follows disease course, including medication information and treatment interventions. Participants' records are continuously updated during their hospital stay and 12 months post-discharge. This pragmatic approach reduces the data collection burden on patients, clinicians, and researchers. | NR | Data collected is collated in a highly secure and encrypted data platform then uploaded to the REACT platform for rapid capture of disease natural history. Clinicians visualize patient trends and identify candidates for intervention studies. Research data is stored anonymously and retained for at least 5 years post-study. | Study data access is restricted to team members via encrypted web services. Anonymized data may be shared internationally with approval from the REACT COVID-19 data access committee. | Data is captured in real-time and backed up weekly, and participants’ records are continuously updated throughout their follow-up period. |
| **Active surveillance with descriptive treatment data** | | | | | | |
| BPSU system [32] | E-reporting card and questionnaire | Data collection forms will be sent to doctors to collect the information from the medical records for each eligible baby, covering pregnancy details, baby characteristics, neonatal management, and outcomes. Reporters not returning forms will be contacted via email after notification. Hospital-based research nurses from the NIHR will support data collection following the study's adoption as an urgent public health priority. | Notifications and completed data collection sheets will be returned via the secure NHS.net email system. [40] | Identifiable and clinical data will be stored separately with a unique ID for each baby. An Open Clinical database will be established, and anonymized datasets will be extracted for analysis. The investigator team will review incoming cases weekly to identify early signals. Data will be retained for five years after the study's completion. [40] | Authorized representatives from the Sponsor and host institution will have direct access for study monitoring and audit. Weekly identified signals will be communicated to relevant governments and organizations to support policy and clinical decision-making promptly. [40] | Weekly electronic BPSU reporting cards notify eligible cases. Monthly BPSU cards confirm cases. Investigator team reviews incoming cases weekly for early signals from surveillance. [40] |
| CNISP program [33] | Standardized case report form and questionnaire | Experienced and trained hospital staff reviewed patient records using a standardized case report form to collect patient’s data of demographic and clinical characteristics, treatment, and outcome. A detailed patient questionnaire was completed via chart review and reported to Public Health Agency of Canada (PHAC). [41] | Data submitted to PHAC via Canadian Network for Public Health Intelligence, a secure online platform. A weekly aggregate data report was submitted electronically to CNISP. | Data was cleaned and verified by PHAC epidemiologists. Inconsistencies were verified by the submitting hospital. | PHAC and site investigators reviewed data-sharing requests. CNISP hospitals received weekly aggregate report by age and regional or provincial level data and a monthly national report from the patient questionnaires. | Real-time hospital-based surveillance system provided timely data, weekly aggregate and monthly descriptive reports. |
| UKOSS system [34] | E-reporting card and E-data collection form | Obstetric consultants (obstetrician, midwife, anaesthetist, perinatal risk management co-ordinator) nominated in each hospital receive monthly report cards from UKOSS. [42] Upon receiving case reports, UKOSS central team requests clinicians to complete electronic data collection forms for detailed information. Data includes women's characteristics, diagnosis, management, outcomes, and therapy details. Maternal and perinatal death data is cross-checked. Research midwives and nurses from NIHR facilitate data collection. Clinicians are contacted by telephone if no response is received after three weeks. | NR | The data collected is anonymous with unique UKOSS numbers to identify patients. Guidance on managing pregnant women with pandemic influenza or novel coronavirus in pregnancy, informed by ongoing data analysis, is produced and reviewed monthly with relevant organizations. | The collected data undergoes analysis and is reported through quarterly newsletters, an annual report, and peer-reviewed publications.[36] Dissemination to women, families, and healthcare practitioners occurs via social media, the program website, and summary articles for professional and third-sector organizations. | Monthly report card and guidance on pregnant women's management, quarterly newsletters, an annual report, and peer-reviewed publication. |

ADE, adverse drug event; ADR, adverse drug reaction; AS, Active surveillance; AZI, azithromycin; BPSU, British Paediatric Surveillance Unit; CADRs, cardiac adverse drug reactions; CMR, computerized medical record; CNISP, Canadian Nosocomial Infection Surveillance Program; EAVE II, Early Pandemic Evaluation and Enhanced Surveillance of COVID-19; ECG, Electrocardiogram; EHR, Electronic health record; EMR, electronic medical record; FDA, Food and Drug Administration; FPVD, French PharmacoVigilance Database; HCA, Hospital Corporation of America; HCQ, hydroxychloroquine; LPVr, lopinavir/ritonavir; ICSRs, Individual Case Safety Reports; INFARMED, Portuguese National Authority for Medicines and Health Products; NHS, National Health Service; NIHR, National Institute for Health Research; NPS, National Pharmacovigilance System; NR, not reported; ORCHID, Oxford Royal College of General Practitioners Clinical Informatics Digital Hub; PHAC, Public Health Agency of Canada; PIDM, Programme for International Drug Monitoring; RCGP RSC, Oxford Royal College of General Practitioners Research and Surveillance Centre; REACT, Research Evaluation Alongside Clinical Treatment; SAE, Serious adverse event; UK, United Kingdom; UKOSS, UK Obstetric Surveillance System; UMC, Uppsala Monitoring Center; US, United States; Vigibase, WHO global database of individual case safety reports; WHO, World Health Organization

**Supplementary 4 - Examples of the studies applying the thirteen active surveillance systems**

| **AS systems** | **Publication date** | **Type of example study** | **Study objectives** | **Setting** | **Period of surveillance** | **Patient population** | **Health products for COVID-19** | **Outcomes of interest** |  |
| --- | --- | --- | --- | --- | --- | --- | --- | --- | --- |
| **Active surveillance on safety** | | | | | | | | | |
| WHO PIDM program [20,21] | 2021 | Retrospective case–noncase study [20] | A pharmacovigilance analysis is conducted on the WHO global database VigiBase to assess a potential signal of acute renal failure linked to remdesivir (REM) in postmarketing databases. | NR | January 1 to August 30, 2020 | COVID-19 patients with case safety reports in VigiBase | REM | Acute kidney injury (AKI), tubular necrosis, kidney disorder, early discontinuation due to kidney disorder |  |
| French pharmacovigilance network [22,23] | 2021 | Retrospective comparative observational study [22] | To compare notifications of HCQ CADRs and their incidences through spontaneous postmarketing reporting before the COVID-19 period in SLE and RA with those reported during the epidemic in COVID-19. | COVID group: off-label use for patients with COVID-19 | COVID group: March 25 to May 25, 2020 | All postmarketing CADRs associated with HCQ, including patients with COVID-19 and treated by HCQ | HCQ | Repolarization and ventricular rhythm disorders, sinus bradycardias, incidence of CADRs |  |
| NPS system [24] | 2021 | Prospective observational study [24] | To evaluate ADRs attributed to either REM or HCQ in patients hospitalised for COVID-19 in Centro Hospitalar de Lisboa cidental. | Hospital | March 16 to August 15, 2020 | Patients with COVID-19 and treated with either REM or HCQ alone or in combination with other medication | REM,  HCQ | ADR cumulative incidence, drug discontinuation, causality assessment |  |
| SafetyWatch system [25] | 2020 | Prospective study of individual case safety reports [25] | To describe the pattern of spontaneous ADR reports from healthcare professionals in patients with SARSCoV-2 in Ghana and lessons learned especially for low- and middle-income nations. | NR | April 1 to July 31, 2020 | SARS-CoV-2 positive patients and on treatment with any medication | Doxycycline, chloroquine, HCQ, AZI, methylprednisolone, LPVr | ADRs,  serious ADRs, causality assessment of ADRs |  |
| PharmaCheck system [26] | 2021 | Prospective observational study [26] | To screen high-risk situations for ADEs, focusing on LPVr and HCQ prescriptions with contraindications or deviations from institutional guidelines. | Hospital | March 24 to May 12, 2020 | All patients with COVID-19 admitted to internal medicine wards with current LPVr or a history of HCQ or both | LPVr,  HCQ | Alert and triggers, recommendations for therapeutic optimisation or additional monitoring |  |
| CEM method [27] | 2022 | Observational prospective single-arm cohort study (a master cohort event monitoring protocol) [27] | Primary objective: To characterize and estimate the incidence of all adverse events (AEs, SAEs, medication errors, off-label use, and misuse) in enrolled patients.  Secondary objectives: 1. to characterize and estimate the incidence of maternal and perinatal outcomes in women inadvertently exposed to molnupiravir during pregnancy and neonate/infant/child exposed during breastfeeding. 2. to detect signals of drug-drug interactions and interactions with traditional medicines. 3. to estimate the incidence of severe COVID-19 disease following treatment with molnupiravir, to detect possible lack of adherence to treatment or lack of effect. | Health facility sites where molnupiravir is provided | Administering molnupiravir from the first authorized dose until three months after the last dose, or  until the end of pregnancy, and the age of 12 months for their children. | Patients with mild to moderate COVID-19 infection and treated by molnupiravir at sites participating in this study in low and middle-income countries | Molnupiravir | All AEs,  SAEs,  maternal and perinatal outcomes events |  |
| **Active surveillance on safety and effectiveness** | | | | | | | | | |
| FDA's sentinel system [28] | 2021 | Surveillance report of COVID-19 [28] | Leveraging Sentinel's pandemic preparation, a surveillance report on COVID-19 census and clinical characteristics in HCA Healthcare was developed to monitor care patterns and clinical outcomes. | Hospital | February 20, 2020-January 10, 2021 | Hospitalized patients with COVID-19 diagnoses (including pregnant women and children) | Dexamethasone, REM, heparin, low molecular weight heparin, monoclonal antibodies | ICU stay,  discharge status,  death,  mechanical ventilation or ECMO |  |
| EAVE II surveillance [29] | 2020 | Prospective observational cohort study  (protocol) [29] | To monitor the daily/weekly progress of the COVID-19 epidemic and evaluate the effectiveness and safety of therapeutic interventions in about 5.4 million individuals registered in general practices across Scotland. | General practice | NR | All residents in Scotland registered with a general practice | Existing or new therapies and antimicrobial medication against COVID-19 | Effectiveness and safety |  |
| ORCHID digital hub [30] | 2020 | Protocol to develop extended COVID-19 surveillance platform [30] | To provides near-real-time data on clinical diagnoses of respiratory infections, including COVID-19 cases and related hospital admissions at participating practices. | Primary care and secondary care | NR | Patients with suspected and confirmed COVID-19 | Concurrent medication that may influence COVID-19 outcomes (e.g., ACE inhibitors, ibuprofen) | Hospital admission,  ICU admission, mechanical ventilation, death related to suspected COVID-19, consumption of antibiotics,  positive COVID-19 test |  |
| **Active surveillance on effectiveness** | | | | | | | | | |
| REACT COVID-19 platform [31] | 2020 | Prospective observational study [31] (ongoing) | To assess the efficacy* of current best practice management strategies and identify subgroups of patients for novel treatment strategies in the form of independent formal randomised control trials. | Tertiary University Hospital | March 7, 2020 to no time-defined end | All patients under the care of UHSFT who are tested or treated for SARS-CoV-2 | Antiviral agent, antibiotic, corticosteroid, ACE inhibitor/ARB, Medication changes for clinic follow up | Death, alive at discharge, palliative discharge, secondary diagnosis (e.g., ARDS), invasive ventilation,  medication changes for COVID-19 complications |  |
| **Active surveillance with descriptive treatment data** | | | | | | | | | |
| BPSU system [32] | 2020 | National prospective cohort study using BPSU [32] (ongoing) | The study aims to describe the incidence, characteristics, transmission, and outcomes of SARS-CoV-2 infection in newborns hospitalized in UK within the first 28 days of birth, informing policies and guidance for healthcare providers, pregnant women and parents. Specifically, it focuses on clinical treatments, including fever and other complication management, antiviral and other COVID-19-specific therapies during and after hospitalization. [40] | Hospital | March 1 to April 30, 2020 (Protocol planned: April 1st, 2020 - April 31st, 2021) | Babies with confirmed SARS-CoV-2 infection in the first 28 days of life who received inpatient hospital care in the UK | Antibiotics,  antivirals, corticosteroids, anti-arrhythmic treatment, immunoglobulin | Length of stay in hospital, still admitted, discharged home, transferred to another hospital, death |  |
| CNISP program [33] | 2020 | Prospective sentinel surveillance [33] (ongoing) | To describe the epidemiology of hospitalized patients with laboratory-confirmed COVID-19 through surveillance in a national network of Canadian acute care hospitals. | Hospital | March 1 to August 31, 2020 (protocol: Year-round seasonal surveillance, July 1 to June 30) [41] | Patients of any age who were admitted to a CNISP hospital within 14 days of a positive SARS-CoV-2 test result (including pregnant women and children) | Antimicrobial (e.g., AZI), antivirals (e.g., Oseltamivir), HCQ, Corticosteroids | Admission to ICU, mechanical ventilation,  all-cause mortality, attributable mortality,  discharge status (discharged, transferred, or still in hospital) |  |
| UKOSS system [34] | 2020 | Prospective national population-based cohort study using UKOSS [34] (ongoing) | To describe the characteristics and outcomes of pregnant women admitted to hospitals with SARS-CoV-2 in the UK to inform ongoing guidance and management. | Hospital | March 1 to April 14, 2020 (protocol: 1st March 2020 – 31st March 2022) [43] | Pregnant women admitted to hospital with confirmed SARS-CoV-2 infection in UK | Antivirals (oseltamivir, LPVr, and REM),  corticosteroids | Incidence of maternal hospital admission and infant infection, rates of maternal death, admission to level 3 critical care unit, fetal loss, caesarean birth, preterm birth, stillbirth, early neonatal death, neonatal unit admission, discharge status (well or still in hospital), and overall mortality. |  |

ACEI/ARB, Angiotensin Converting Enzyme Inhibitor/Angiotensin Receptor Blocker; ADE, adverse drug event; ADR, adverse drug reaction; AE, adverse event; AS, Active surveillance; AZI, azithromycin; BPSU, British Paediatric Surveillance Unit; CADR, cardiac adverse drug reaction; CNISP, Canadian Nosocomial Infection Surveillance Program; EAVE II, Early Pandemic Evaluation and Enhanced Surveillance of COVID-19; ECMO, Extracorporeal membrane oxygenation; FDA, Food and Drug Administration; HCA, Hospital Corporation of America; HCQ, hydroxychloroquine; ICU, Intensive care unit; LPVr, lopinavir/ritonavir; NPS, National Pharmacovigilance System; NR, not reported; ORCHID, Oxford Royal College of General Practitioners Clinical Informatics Digital Hub; PIDM, Programme for International Drug Monitoring; RA, rheumatoid arthritis; REM, remdesivir; SAE, Serious adverse event; SLE, systemic lupus erythematosus; UHSFT, University Hospital Southampton NHS Foundation Trust; UK, United Kingdom; UKOSS, UK Obstetric Surveillance System; Vigibase, WHO global database of individual case safety reports; WHO, World Health Organization

*Although efficacy was the term used by the authors for this observational study, it was treated as effectiveness in this review.

**Supplementary 5 – Security and quality of the thirteen active surveillance systems**

| **AS systems** | **Privacy and security of data** | **Quality of data** | **Strengths** | **Limitations** |
| --- | --- | --- | --- | --- |
| **Active surveillance on safety** | | | | |
| WHO PIDM program [20,21] | The provided information is solely used to enhance products and services, with rigorous measures to ensure data security. Updates to the privacy policy may occur as data protection laws evolve with technology. | VigiBase's automated entry process checks each report against predefined quality criteria. UMC collaborates with member countries to enhance the quality of their ICSRs and offers guidance on its importance in analysis. The vigiMatch method for duplicate detection has been used at UMC for signal detection since 2014. For retrieving and analyzing ADR reports related to COVID-19 treatments, it's essential to code the drug's indication of use with appropriate COVID-19 terms available in MedDRA. [37, 38] | NR | The limitations of the retrospective design of pharmacovigilance analysis include underreporting and residual confounders. |
| French pharmacovigilance network [22,23] | NR | NR | NR | The true incidence of HCQ-induced CADRs may be underestimated, possibly due to surveillance bias. |
| NPS system [24] | NR | NR | NR | A relatively small patient sample was studied, and ADR monitoring was limited to the hospital stay, potentially leading to underreporting. Additionally, no follow-up of reported ADRs was conducted. |
| SafetyWatch system [25] | NR | The availability of reporting forms and the safety monitoring plan informed healthcare workers in treatment centers about pharmacovigilance requirements during the pandemic, enabling them to report safety issues. | NR | The study's limitations include a small number of ADR reports reviewed during the pandemic and underreporting of ADRs in Ghana. |
| PharmaCheck system [26] | NR | NR | NR | Without a control group, it's unclear if the interventions led to fewer ADEs. The system's sensitivity wasn't established; it only detected and analyzed alerts associated with at least one trigger. |
| CEM method [27] | Data will be handled in compliance with all relevant data protection and privacy laws to prevent unauthorized access. Strict regulations will govern data capture, forwarding, processing, and storage. An identification log will be securely stored in the study country, with key-coded data kept in a secure database at the WHO headquarters in Geneva. | Study sites may undergo quality assurance visits. Training will be provided for all staff involved. Automatic quality checks will detect out-of-range or unusual data, where applicable, for all data entered electronically | NR | Long-term adverse events won't be detected with this design. There's a risk of selection bias if some participants don't consent or can't comply with follow-up. Loss to follow-up is possible, especially if patients are hospitalized and can't respond. |
| **Active surveillance on safety and effectiveness** | | | | |
| FDA's sentinel system [28] | Sentinel functions as a privacy-preserving distributed data network, ensuring each organization retains control over its data. Executable computer programs are securely distributed to partners for execution against curated data, with results securely aggregated and analyzed. | Data partners can access full-text medical records to validate electronic data or provide clinical details. Each organization's data undergoes extensive quality assurance checks before analysis approval.  A natural history master protocol guides various COVID-19 studies using claims, EHR, or linked data, promoting shared approaches and definitions for the studies funded by FDA and others. | NR | NR |
| EAVE II surveillance [29] | NR | Regular validation checks are applied to the Scottish Morbidity Record (SMR) database. | The latest data quality assessment of these SMR datasets indicates over 90% completeness and accuracy, consistent with previous years. | NR |
| ORCHID digital hub [30] | ORCHID adheres to data protection laws and NHS Digital Data Security and Privacy Policy. RCGP RSC accessed data via its secure network, restricting cloud access to authorized users who undergo induction. | Systems will offer feedback to member practices on the coding quality to promote improvement change. | NR | NR |
| **Active surveillance on effectiveness** | | | | |
| REACT COVID-19 platform [31] | The collected database documents (paper and electronic) are securely stored in compliance with the general data protection regulations of 2018 throughout and after the trial. | Most data capture is electronic, minimizing missing data. Collected data aligns seamlessly with routine clinical care for COVID-19 patients. | The system's strength lies in capturing the detailed longitudinal path of clinical care without a strict visit protocol to follow. | The system's capability to capture detailed longitudinal clinical care paths may lead to more incomplete datasets, as some patients may have more available data. |
| **Active surveillance with descriptive treatment data** | | | | |
| BPSU system [32] | The study adheres to general data protection regulations and the data protection act 2018, requiring prompt de-identification of data. Electronic document storage will restrict access to study staff and authorized personnel only. Staff are committed to safeguarding participant privacy and receive ongoing security training. [40] | The collected data will include identifiers for cross-checking with other sources, de-duplication, and later linkage for additional treatment details and outcomes. Data will be double entered an Excel database. Monitoring or audits will follow the approved protocol, GCP, regulations, and SOPs. [40] | This study's strength lies in reporting population-level incidence data across the UK through an established active surveillance system with high reporting levels by UK pediatricians during the study period. The cases identified through other sources underscore the importance of multiple-source notification and linkage during a health crisis. | The study limitations include focusing on essential data to reduce reporting burden on pediatricians in the coordinated national health service response to the COVID-19 pandemic, resulting in the absence of detailed longitudinal physiological, biochemical, or hematological test data. |
| CNISP program [33] | A secure online platform facilitated data submission through the Canadian Network for Public Health Intelligence | NR | NR | Data collection was limited to information from patient charts. Analyses were descriptive in nature. |
| UKOSS system [34] | Data will be securely stored on a university network, accessible only to authorized researchers and responsible University members. Access by any other individuals will require the review by the UKOSS Steering Committee and Research Ethics Committee. [43] | Data from women giving birth in the UK two years prior to any future pandemic, not infected with influenza or novel coronavirus, will be used as a historical comparison cohort to minimize potential bias due to service changes. [43] | This study demonstrates the effectiveness of systems like UKOSS, swiftly activated for comprehensive population-based studies during public health emergencies. The NIHR’s clinical research network, supported by midwifery and obstetric leads, facilitated rapid and accurate data collection, even amidst pandemic-related health system pressures | The data collected for this rapid national cohort study focused on essential items, omitting daily indicators of women's clinical condition or test results. |

ADE, adverse drug event; ADR, adverse drug reaction; AS, Active surveillance; BPSU, British Paediatric Surveillance Unit; CADR, cardiac adverse drug reaction; CEM, Cohort Event Monitoring; CNISP, Canadian Nosocomial Infection Surveillance Program; EAVE II, Early Pandemic Evaluation and Enhanced Surveillance of COVID-19; EHR, Electronic health record; FDA, Food and Drug Administration; GCP, good clinical practices; HCQ, hydroxychloroquine; ICSR, Individual case safety report; NHS, National Health Service; NIHR, National Institute for Health Research; NPS, National Pharmacovigilance System; NR, not reported; ORCHID, Oxford Royal College of General Practitioners Clinical Informatics Digital Hub; PIDM, Programme for International Drug Monitoring; RCGP RSC, Oxford Royal College of General Practitioners Research and Surveillance Centre; REACT, Research Evaluation Alongside Clinical Treatment; SMR, Scottish Morbidity Record; SOP, standard operating procedures; UK, United Kingdom; UKOSS, UK Obstetric Surveillance System; UMC, Uppsala Monitoring Center; Vigibase, WHO global database of individual case safety reports; WHO, World Health Organization
